# Machine learning assisted exploration of the influential parameters on the PLGA nanoparticles

**DOI:** 10.1038/s41598-023-50876-w

**Published:** 2024-01-11

**Authors:** Sima Rezvantalab, Sara Mihandoost, Masoumeh Rezaiee

**Affiliations:** 1 Chemical Engineering Department, Urmia University of Technology, Urmia, 57166‑419 Iran; 2 Present Address: Electrical Engineering Department, Urmia University of Technology, Urmia, 57166‑419, Iran

**Keywords:** Biomedical materials, Pharmaceutics

## Abstract

Poly (lactic-co-glycolic acid) (PLGA)-based nanoparticles (NPs) are widely investigated as drug delivery systems. However, despite the numerous reviews and research papers discussing various physicochemical and technical properties that affect NP size and drug loading characteristics, predicting the influential features remains difficult. In the present study, we employed four different machine learning (ML) techniques to create ML models using effective parameters related to NP size, encapsulation efficiency (E.E.%), and drug loading (D.L.%). These parameters were extracted from the different literature. Least Absolute Shrinkage and Selection Operator was used to investigate the input parameters and identify the most influential features (descriptors). Initially, ML models were trained and validated using tenfold validation methods, and subsequently, next their performances were evaluated and compared in terms of absolute error, mean absolute, error and R-square. After comparing the performance of different ML models, we decided to use support vector regression for predicting the size and E.E.% and random forest for predicting the D.L.% of PLGA-based NPs. Furthermore, we investigated the interactions between these target variables using ML methods and found that size and E.E.% are interrelated, while D.L.% shows no significant relationship with the other targets. Among these variables, E.E.% was identified as the most influential parameter affecting the NPs' size. Additionally, we found that certain physicochemical properties of PLGA, including molecular weight (Mw) and the lactide-to-glycolide (LA/GA) ratio, are the most determining features for E.E.% and D.L.% of the final NPs, respectively.

## Introduction

Nanomedicine, the science of designing and synthesizing medicines and imaging agents at the nanoscale, has garnered significant attention from the scientific community in recent decades. This field encompasses a wide array of materials that can serve as effective delivery platforms^1–3^. Since the beginning of the nanomedicine field, scientists from various backgrounds have tried to harness diverse knowledge to impel the advancements in the design of efficient nano-scaled pharmaceuticals. To boost the efficacy of nanomedicines and expedite their clinical application, a variety of strategies have been experimented with: co-loading of drugs and therapeutic agents (immunotherapy^4,5^, phototherapy^6,7^, imaging agents^8,9^, etc*.*), surface modification, and functionalizing with moieties (targeting ligands^10^, proteins^11,12^, etc*.*). Experimentation and traditional trial-and-error practices are common, costly, and irregular in many of the strategies mentioned. ML-based methods, however, have recently gained prominence in the analysis and evaluation of drug delivery systems^13^.

A variety of computational methods were utilized in this field, including quantum mechanics (at the electron, atomic, and molecular scales), molecular dynamics simulations (atoms and molecules moving at nanoscales), and mathematical and physiological pharmacokinetic/pharmacodynamic modeling (physicochemical behavior of drug delivery systems). Additionally, artificial intelligence (AI) based methods, including machine learning (ML) and deep learning have recently come into focus in this field, paving the way for data-driven nanomedicine. In comparison with other methods that are mostly based on theory, data-driven nanomedicine has gained a reputation for being based on experimental data as well as mathematical modeling and evaluations. Unlike other computational approaches, AI methods do not have a scale limit and can be applied to a wide range of applications. As an example, AI has been applied to the design of small drug molecules^14,15^, as well as the control of physicochemical properties of formulations (*e.g.* size, shape, drug loading efficiency, etc.) and even evaluation and optimization of (pre-)clinical data^16,17^. It is for this reason that these methods can help scientists cope with the complexity and ambiguity of practical DDSs, including polymer-based systems.

Its abilities to load and deliver a broad range of therapeutic and theranostic agents have made PLGA a preferred polymer-based DDS^18,19^. Due to its natural and non-toxic byproducts of PLGA degradation and its ability to conjugate various ligands, it has been approved as a DDS for a variety of formulations, including Decapeptyl® and several other microparticles based on PLGA, PGA, and PLA^20,21^. A wide range of therapeutic agents have been successfully delivered by PLGA-based (NPs), but factors that affect their physicochemical properties are complex and intertwined. For instance, Fredenberg et al.^22^ discussed the complexity of the drug release mechanism and physicochemical properties of PLGA-based NPs. In addition to drug release mechanisms, various parameters can impact PLGA-based NPs' final performance and application. Previously, the determinant parameters and the complexity between them were portrayed as a Rubik's cube, where a small change in one brick (parameter) would affect the whole plane (other parameters and subsequent outcomes). NP size, drug loading and release are all influenced by the molecular weight (Mw) of the polymer, LA/GA ratio, solvent, and the synthesis method^23^.

Since many parameters are ignored in traditional mathematical methods to avoid complexity, it appears impossible to establish a clear relationship between these effective parameters and their outcomes. For example, diffusion-driven models of drug-carrier interactions and diffusion-driven theoretical simulations of drug loading and release are limited by many assumptions. In this type of simulation, polymer segments are ignored due to the model complexity and treated as one polymer mass. These mathematical models also require approximate initial and boundary conditions. It is possible, however, to add each polymer's contribution to ML-based methods, and get a deeper understanding of their interactions. This type of method can provide more accurate and detailed information about material behavior, which can be used to predict the behavior of more complex systems based on the properties of the material. In this regard, using AI-assisted methods it is possible to design formulations with better control of properties and optimal outcomes^24–26^. For instance, multilayer perceptron artificial neural networks (ANNs) were used by Damiati et al.^27^ to determine the sizes of (single or multiple) PLGA microparticles produced in microfluidic systems. PLGA solution concentration, microfluidic device geometry, and organic and aqueous flow rates have been used as input layers (effective factors) to train the ANN method. In their study, they reported highly accurate size predictions through microfluidics in the generation of PLGA-based microparticles. The same multilayer perceptron ANNs were used in a subsequent study to optimize indomethacin-loaded PLGA microparticle properties^28^. A successful application of the AI method was reported in the design of microparticles with satisfactory D.L.% and E.E.% (7.79 and 62.35%, respectively).

In this study, through the application of ML methods, we seek to advance our understanding of the critical factors involved in the synthesis and characteristics of PLGA-based NPs. Size, E.E.%, and D.L.% of the final NPs are influenced not only by polymer properties (such as LA/GA ratios, Mw, polyethylene glycol (PEG) layer) but also by numerous process related properties like synthesis methods (including nanoprecipitation, single or double emulsion, microfluidically), drug type, and of course solvents and presence of surfactants in the synthesis section. We collected all the essential information from the literature on PLGA-based NPs through data mining techniques. We evaluated the performance of various regression models on the data and also determined the order of importance of different features for every target. Additionally, we assessed the interactions between size, E.E.%, and D.L.% using ML techniques. At the end, we modeled data utilizing the effective features and the selected regression algorithm.

## Results and discussion

**Figure 1:**
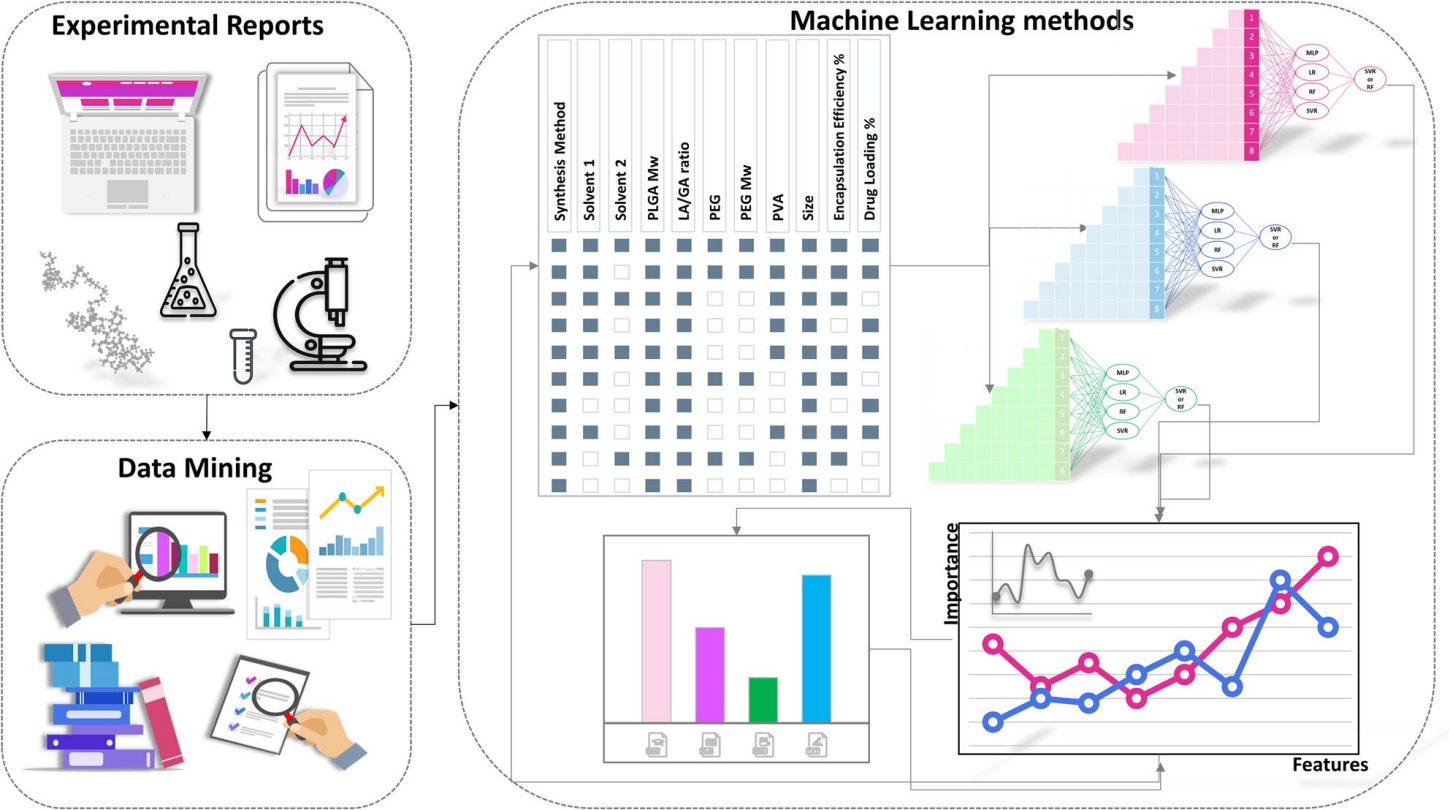
An overview of the current study’s workflow. The reported data from experimental researches has been collected through a data mining stage. After a data cleaning step, ML-based algorithms have been employed to predict the effective parameters in the final characteristics of PLGA-based NPs.

With the ML method, we aimed to recognize a relationship between multiple characteristics so that future investigations could be guided accordingly. In this regard, a database of PLGA-based NPs has been constructed that covers a broad range of information about the polymer chemistry and NPs’ preparation method. Figure 1 outlines the main workflow of the current study.

The dataset is constructed with data from over a hundred research articles using keyword “PLGA NPs” which the detailed information was gathered all the reported factors. Eight features were identified as influential features in the final properties and performances of NPs. In this study, we selected the NPs' size, E.E.%, and D.L.% as significant parameters that can have a significant impact on formulation performance and therapeutic effect. Here are the definitions and measurements of E.E.% and D.L.% used in experimental studies:

1 $$E \cdot E\cdot \mathrm{\%}=\frac{\mathrm{Weight \; of \; entrapped \; drug \; in \; NPs}}{\mathrm{Weight \; of \; total \; added \; drugs}}\times 100$$

2 $$D\cdot L\cdot \mathrm{\%}=\frac{\mathrm{Weight \; of \; drug \; content \; in \; NPs}}{\mathrm{Weight \; of \; NPs}}\times 100$$

A number of features are polymer-related, for example, PLGA Mw, the presence of PEG in the formulation, and PEG Mw have the ability to influence the final properties of NPs. It worth mentioning that polymers' molecular weight has been used as an average of reported molecular weights. In addition, factors related to the preparation stage of NPs include the NPs preparation method (hereafter called the method), the solvents, and the presence of PVA (hereinafter called PVA). Data mining revealed that nanoprecipitation, single/double emulsion, and microfluidics methods are reported for the synthesis of NPs. Solvents used to dissolve PLGA polymers along with drugs to be loaded are another important aspect of NPs preparation that can affect final characteristics and performance. The literature review reports the use of numerous solvents in this process.

### Feature and model selection

**Figure 2:**
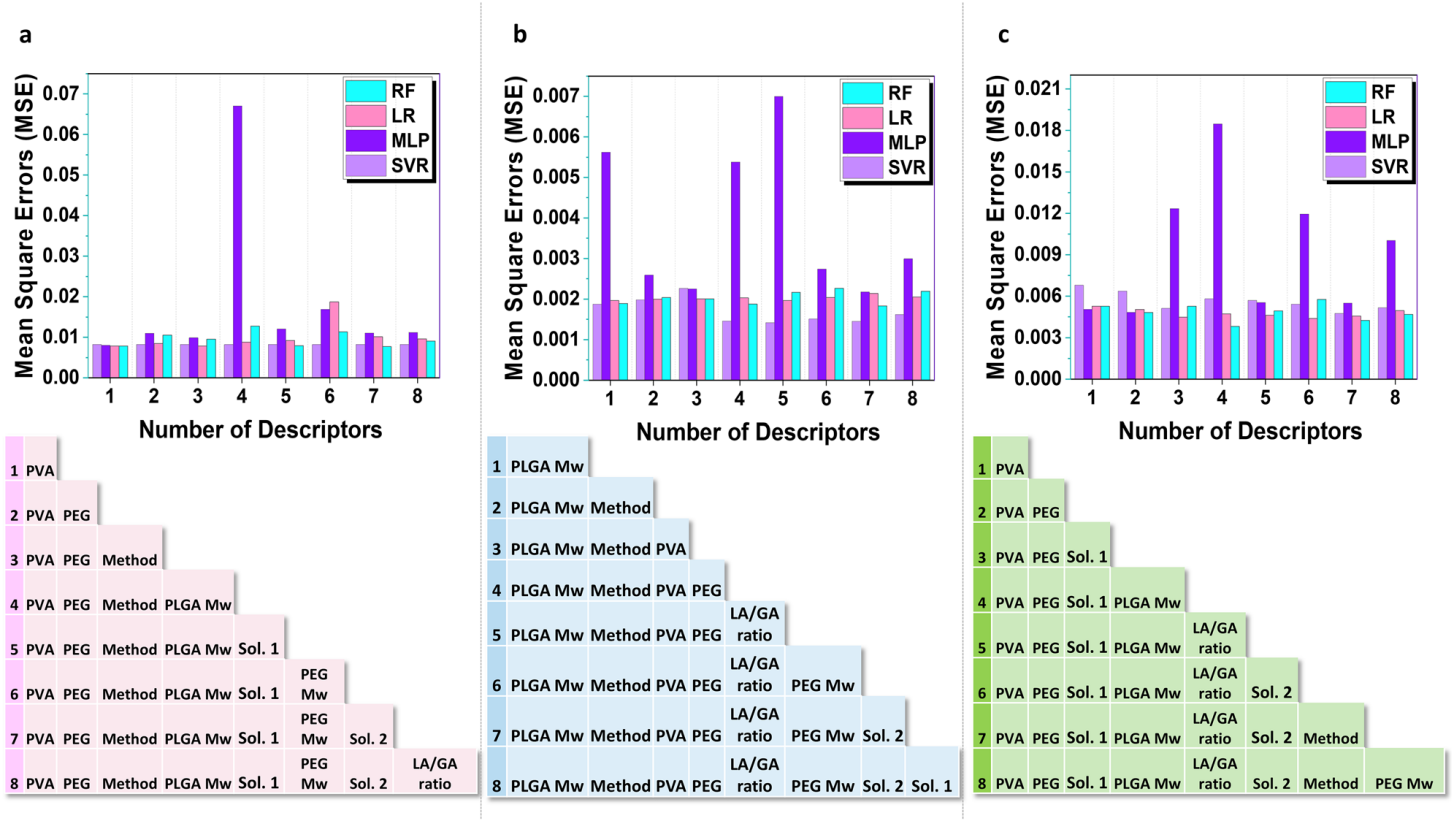
(**a**–**c**) Summary of the performance of various ML models for the prediction of size, E.E.%, and D.L.% using various features, respectively. Mean square errors (MSE) are considered the predictive performance measure in each case. A detailed description of the features and assigned numbers are included in the table under each diagram to avoid clumsiness in plots on the x-axis.

After compiling the dataset, our primary objective was to recognize and analyze the relationships within the gathered data. To achieve this, we employed a variety of well-established ML methods, including RF, SVR, LR, and MLP, and systematically applied them to the dataset. The hyperparameters of each ML model were carefully adjusted during the training and validation phases, utilizing a tenfold validation technique for each modelFurthermore, to evaluate the significance of different features (descriptors) and obtain a comprehensive overview of each descriptor’s impact on the target and sort them by their weights, the feature set and target were subjected to the Lasso algorithm to assign a coefficient weight to each feature, as documented in Table 1. Subsequently, these coefficients were sorted and sequentially applied to the various ML algorithms, allowing us to compare their performance in terms of MSE, as illustrated in Fig. 2. The results obtained for size and E.E.% demonstrate that SVR consistently yields lower MSE across various feature numbers, outperforming other algorithms (Fig. 2a,b). In the case of D.L.%, although SVR performed reasonably well, LR exhibited the lowest MSE across different feature numbers, as shown in Fig. 2C.

**Table 1:** Coefficient weight of each feature computed using Lasso algorithm.

	Size	E.E.%	D.L.%
Method	0.23333	0.12025	0.13114
Solvent 1	0.05756	0	0.24862
Solvent 2	0.01376	0.0038	0.11565
PLGA Mw	0.13763	0.14582	0.27038
LA/GA ratio	0	0.05446	0.47369
PEG	0.27168	0.03584	0.0767
PEG Mw	0.12104	0.02146	0
PVA	0.22702	0.03216	0.43626

Additionally, Fig. 2 illustrates the impact of different features on model performance. In the case of size (Fig. 2a), it is evident that when utilizing 7 features, which includes all features except LA/GA ratio, most ML models achieve the lowest level of MSE. Similarly, for E.E.%, all algorithms exhibit minimal error when considering 7 features, encompassing all features except Solvent 1. Regarding D.L%, Fig. 2c highlights that nearly all ML algorithms perform optimally with 7 features, which include all features except PEG Mw.

General view of results shows that the SVR model had the lowest MSE for size, E.E.%, while LR had best performance for D.L.%. The performance of SVR was with seven features *the* best among other ML models with the lowest MSE (0.0082 ± 6.48 × 10^–6^) and (0.001696 ± 0.00029) respectively. In the case of D.L.%, LR had the best performance with six features, however; other methods such as MLP had the high MSE. Further scrutinizing showed that LR with seven features has the minimum MSE (0.00486 ± 0.00058) whereas other algorithms also were in the low MSEs.

**Figure 3:**
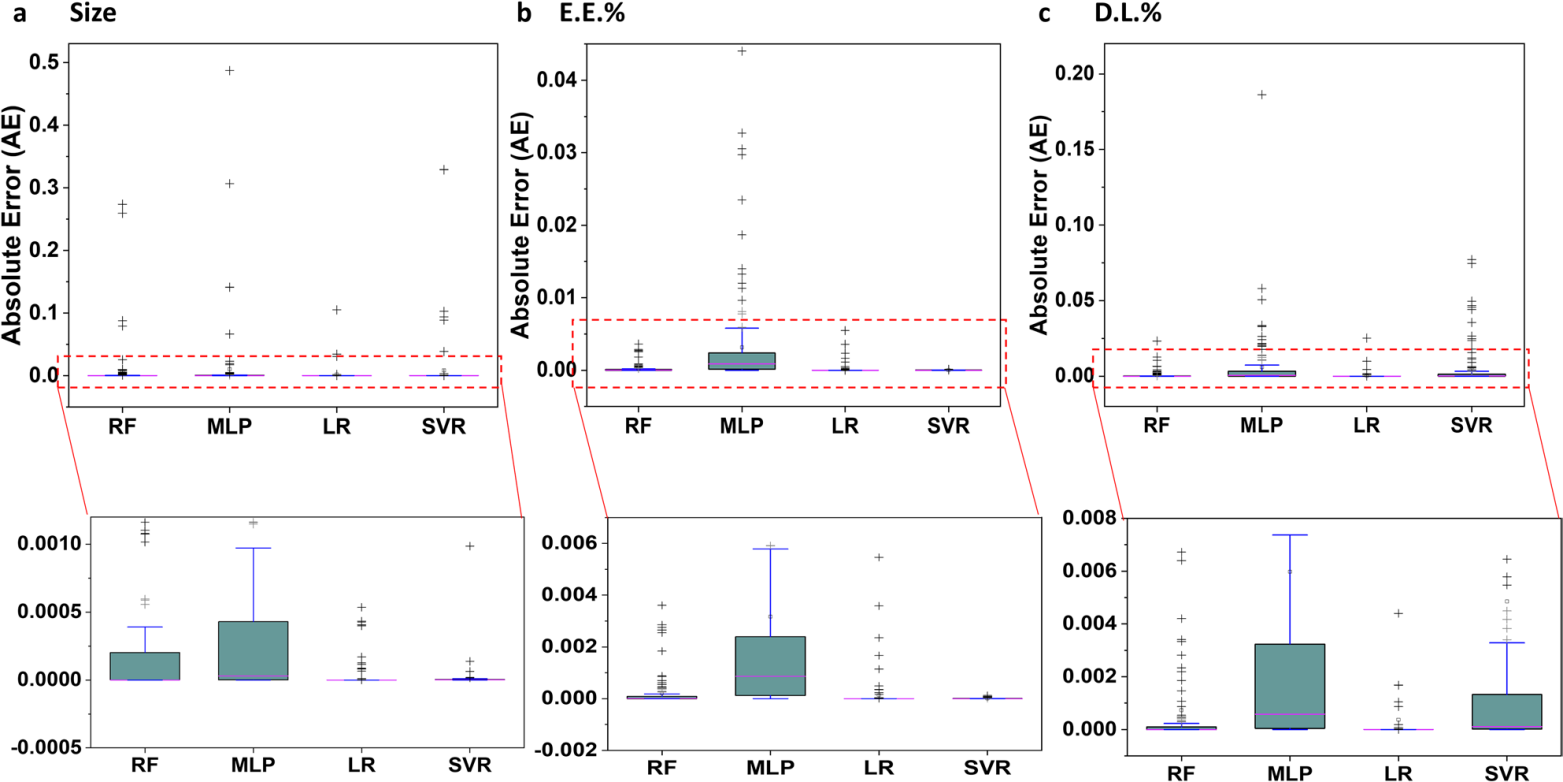
Validation of various ML models' performance based on absolute error for the selected sets of features. Subfigure (**a**) displays the absolute errors (AE) obtained with different ML models for the 7th set of feature descriptors used to predict size. Subfigure (**b**) demonstrates the AE obtained with different ML models for the 7th set of feature descriptors in predicting E.E.%. Lastly, Subfigure (**c**) exhibits the AE obtained with different ML models for the 7th set of feature descriptors in predicting D.L.%. To enhance the comparison of AE values, red lines are used to represent zoomed-in AE values.

Furthermore, we conducted an additional experiment to validate the model selection. After determining the number of features for the models in the previous experiment, these selected features were applied to all ML algorithms to predict the respective targets, and Absolute Errors (AEs) were calculated. This experiment was carried out separately for size, E.E.%, and D.L.%. Figure 3 provides a summary of the results for each target, confirming the accuracy of the selected techniques in the previous experiment. For size and E.E%, the SVR method exhibited the lowest AE, as indicated by the smaller candle size and proximity to zero. In the case of D.L.%, LR demonstrated the lowest AE, as reflected in the candle size and proximity to zero.

### Assessing the interactions between different targets in the presence of the selected features

In this phase, we examined the interactions between different targets in the presence of the selected features. We utilized the LASSO algorithm to evaluate these interactions. For each target, we applied the LASSO algorithm to the 7th set of features (depicted in Fig. 2) along with the other two targets. An intriguing aspect of our approach was considering the influence of each target on the others. We hypothesized that size, E.E.%, and D.L.% exhibit interdependencies. For example, changes in E.E.% may impact the size of NPs, and vice versa. Consequently, when evaluating each target, we also took into account the other targets as input features.

**Figure 4:**
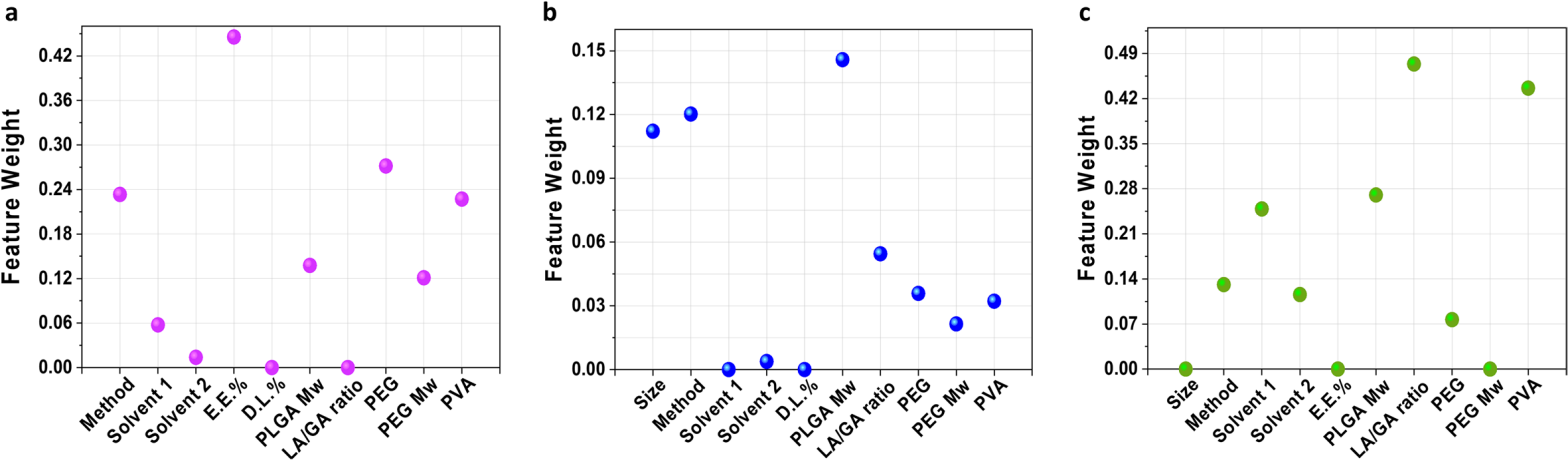
Effective features on the NPs’ size. (**a**–**c**) Impact of various features on the size, E.E.%, and D.L.%, respectively.

Figure 4 provides an overview of the impact of all features on the size, E.E.%, and D.L.%, allowing for a comparison of their respective weights. Notably, E.E.% demonstrates the most significant influence on the size of the NPs, confirming the accuracy of our previous assumption, as depicted in Fig. 4a. Additionally, the presence of a PEG layer surrounding PLGA-based NPs is observed to have a crucial effect on their size, which has been well-documented in several studies^29–31^. For instance, previous research by Afshari et al.^32^, found that PLGA NPs with PEG layers exhibited smaller sizes compared to bare PLGA NPs.

Additionally, the synthesis method as well as presence of PVA during NP production, can be very influential, according to the plot. Reports on the impact of PVA on the size of PLGA-based NPs are contradictory; some report a decrease^33,34^ and others an increase^35,36^. Nevertheless, our findings are in good agreement with previous reports that confirm the NPs sizes are dependent on the presence of PVA. Furthermore, another effective descriptor as a synthesis method. Finally, it can be understood that Mw of both (PEG and PLGA) polymers are effective in determining the size of NPs. Based on a library of NPs produced by both PLGA and PEG polymers, both polymers have a significant impact on the final NP size^37,38^. Altogether, it is imperative that polymer physicochemical properties as well as synthesis methods be taken into consideration when designing and producing PLGA-based NPs.

In the subsequent step, we repeated this experiment using LASSO for the prediction of E.E.%. We observed that LASSO model identified the size among effective features on the E.E.%, according to Fig. 4b. Additionally, the classifying model identified that the molecular weight of PLGA plays the biggest role in the importance of features in E.E.%. Our results are in good agreement with previous papers that have reported a correlation between size and E.E.%^39,40^. Furthermore, synthesis method is recognized as effective descriptor in the NPs E.E.%. Interestingly, it can be understood that the synthesis method acts as a double-edged sword that higher E.E.% gives rise into larger sizes. Therefore, feature researchers can design the NPs based on the trade-off between size and E.E.%. There is an interplay between size and E.E.%, as the third important effective descriptor on E.E.%. This observation is parallel with the previous finding and corroborates our hypothesis. Remarkably, analysis of feature weights form size and E.E.% reveals that D.L.% has no contribution in these targets and its weight in both cases is zero. This can be related to the different definitions of E.E.% and D.L.% (Eqs. 1 and 2, respectively). As mentioned it before, E.E.% refers to the proportion of encapsulated drug to the dissolved drug in a solvent. D.L.%, on the other hand, shows the weight of entrapped drugs in NPs over their total weight. Thus, this unexpected outcome was a direct result of the different definitions of E.E.% and D.L.%.

Similar evaluations have been conducted for D.L.% to identify its dependency on features. Firstly, testing the effective features on D.L.% led to the observation that there is no relationship between D.L.% and other targets (size and E.E.%), as expected (Fig. 4c). Additionally, LA/GA ratios can enormously affect D.L.% because, from a practical point of view, LA/GA ratio can alter the hydrophobicity and crystallinity of the polymers. In consequence, it may influence drug interactions with PLGA polymer chains, since it shifts the affinity of a drug for PLGA polymer. Obviously, D.L.% of NPs is also sensitive to the presence of PVA in formulations. In agreement with previous articles^41,42^ and similar to E.E.%, D.L.% also depends on the presence and concentration of PVA. Again, we observe that polymer Mw acts as a double-edged sword in that the highest D.L.% is corresponding to the lowest E.E.%. Concludingly, Mw of polymer chains is one of the characteristics that researchers should take care of and choose according to their target in order to produce NPs without sacrificing one property for another.

**Figure 5:**
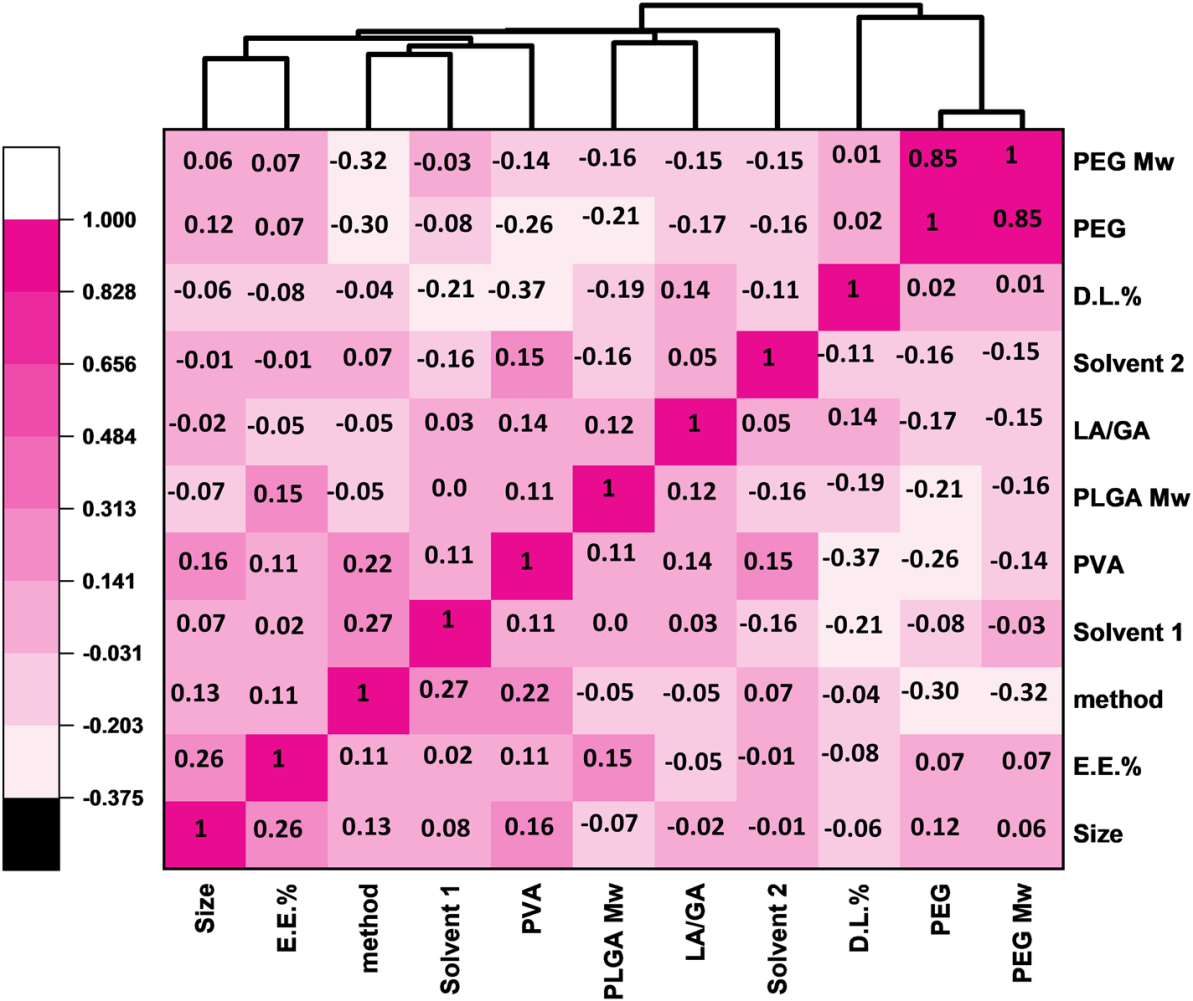
Correlation heatmap of all input features. A dark pink color indicates an absolute correlation (= 1) and a lighter pink color indicates a negative correlation.

Furthermore, to gain a better understanding of the relationship between the selected features and targets, we have prepared Fig. 5, which presents the correlation values between all features and targets. Correlation evaluates the linear relationship between two variables. A correlation value between 0 and 1 indicates a positive linear relationship, while a correlation value between -1 and 0 indicates a negative linear relationship. A correlation value of 0 implies that there is no linear relationship between the two variables. According to Fig. 5, we can investigate the linear relationship between the final feature set and the targets one by one. For example, the PEG and LA/GA ratio exhibit the highest correlation, with a value of 0.85. This indicates that when the PEG increases or decreases, the LA/GA ratio behaves in the same manner. On the other hand, the correlation between PEG Mw and E.E.% is − 0.37, indicating a linear relationship between PEG Mw and E.E.%, but in the opposite direction. Overall, these results suggest that linear correlations of inputs are useful in modeling the targets. However, it's important to note that correlation cannot capture non-linear relationships between variables. Therefore, further investigation is necessary to understand the contributions of individual inputs.

Furthermore, Table S1 outlines effects of each input feature on each target individually in terms of r^2^.

### Model interpretation after descriptor selection

**Figure 6:**
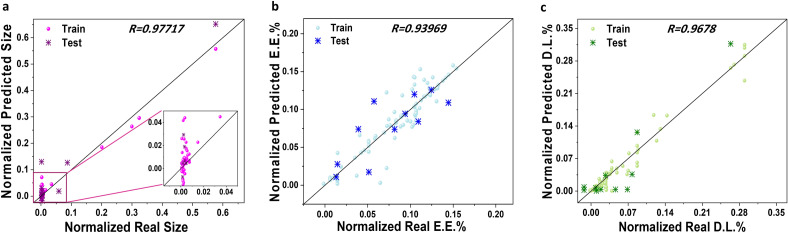
Relationship between the experimentally reported size, E.E.%, and D.L.%, predicted values.

After the model and feature selection process, we focused on interpreting the models using the remaining *features*. In this stage, we removed the features that had zero weights in the previous section and evaluated the models using the non-zero features. Specifically, we excluded the features D.L.% and LA/GA ratio for size, as well as D.L.% and sol1 for E.E.% and size. Additionally, size, E.E.%, and PEG Mw were not considered for the DL case. To ensure consistency, all continuous variables were normalized to the range of 0 to 1. As mentioned earlier, we employed SVR with a radial basis function (RBF) kernel for predicting size and E.E.%, while LR was utilized for D.L.%. For SVR, we set the regularization parameter to 1 and the gamma parameter to 'scale'. In the case of LR, we used the binomial logistic regression model in logit mode. Figure 6 showcases the data for each target, with the selected features applied to the respective ML model. The scatter plots in the figure illustrate the training and testing results. It is evident that our proposed ML algorithm effectively learns from the training data and exhibits accurate predictions during the testing phase.

## Conclusion

In conclusion, with advancements in computer science research trends, we suggest a data-driven approach, to design and produce PLGA-based NPs to be used as efficient DDSs. For this purpose, ML techniques are used to exploit data from previous research articles and enhance the knowledge of algorithms on the relationships between multiple features. Our assessments revealed that the SVR model can be advantageous for the prediction of size and E.E.% of NPs with datasets containing 7 features, while LR showed the best performance with the same number of features for the prediction of D.L.%. Further evaluations revealed that size and E.E.% also interplay and are one of the most effectors among other features.

## Methods

### Data mining

The dataset for the current study was gathered from published articles about PLGA-based NPs. More than 200 studies were found through a search in google scholar using the keywords “PLGA NPs”, “drug delivery”, “drug loading”, and “encapsulation efficiency”. Afterward, reported information about size, E.E.%, D.L.%, presence of PEG and polyvinyl alcohol (PVA) in the formulation and synthesis, LA/GA ratio, Mw of PLGA and PEG, and solvent(s) used in the synthesis method was collected from papers. Table 2 outlines the data that were mined from papers with their numerical distribution. It should be mentioned, the high standard deviations (SD) of data in Table 2 indicates that the data points exhibit significant diversity around the mean. This diversity is important as it helps prevent ML techniques from becoming biased towards specific parts of the data. By having a larger SD, our dataset encompasses a wider range of values, allowing the ML algorithms to capture and learn from the various patterns and variations present in the data. This ultimately enhances the robustness and generalizability of our ML models.

**Table 2:** The information collected from papers.

Input Features		Target variables	
Name	Range	Name	Range
PLGA Mw (kDa)	45.10 ± 38.7	Size (nm)	234.71 ± 180.2
LA/GA ratio	50/50, 65/35, 75/25, 80/20, 85/15, 95/5, 90/10	E.E.%	50.75 ± 26.5
Presence of PVA	0 or 1	D.L.%	16.95 ± 26.7
Presence of PEG	0 or 1		
PEG Mw (kDa)	0.83 ± 1.79		
Solvent 1	As Table 2		
Solvent 2	As Table 2		
Synthesis Method	Nanoprecipitation, Double emulsion, Single emulsion, Microfluidics		

To construct a usable dataset for ML models training, machine-readable features were reported as *PLGA and PEG Mw (as the average of reported Mw range in the papers), LA/GA ratio (75/25, 50/50, 80/20, 65/35, 85/15, 95/5, and 90/10), synthesis method (nanoprecipitation, single emulsion, double emulsion, and microfluidics), presence of PVA and PEG (as a binary digit, one and zero for presence and absence, respectively, in the composition or synthesis method)*, and the list of solvents with their assigned codes, as in Table 3.

**Table 3:** Reported solvents in the papers used for the preparation of PLGA NPs and their assigned readable codes for the ML models.

Solvent	Code	Solvent	Code
Acetone	1	Dimethylsulfoxide	8
Dichloromethane	2	Tetrahydrofuran	9
Acetonitrile	3	Ethyl acetate	10
Chloroform	4	Hydrochloric acid	11
Ethanol	5	Dimethylformamide	12
Methanol	6	p-Dimethyaminobenzaldehyde	13
Sodium oleate	7	Methylene chloride	14

Raw data will be available from the authors upon reasonable request. In this regards, readers can contact corresponding authors Sima Rezvantalab and/or Sara Mihandoost.

### ML-based models

In this study, we utilized various ML-based techniques, including multilayer perceptron (MLP), random forest (RF), logistic regression (LR), and support vector regression (SVR), to predict the size, E.E.%, and D.L.% of PLGA data.

To evaluate the performance of these ML models, we split the data into training and testing sets using a 70/30 ratio. During the training process, we fine-tuned the hyperparameters of each model using the tenfold cross-validation method, which helps in optimizing the model's performance and generalizability. It is worth mentioning that all the ML experiments were conducted using MATLAB 2022.A concise description of each model used is presented as follows:

*MLP*: In this study, we utilized an MLP network equipped with two hidden layers, one with 7 neurons and the other with 14 neurons, incorporating hyperbolic tangent sigmoid and logarithmic sigmoid transfer functions. The determination of the number of neurons in the hidden layers was a result of a trial-and-error process^43^. For training the neural netw *boost* ork in this study, we employed the Levenberg–Marquardt backpropagation method, conducted over 1000 epochs, while considering a minimum performance gradient of 1*e-7.

*RF*: is an ensemble learning method that excels in predicting continuous numerical values. The algorithm builds an ensemble of decision trees and combines their predictions to achieve accurate results. Let's denote the input features as $X=\left\{{X}_{1}.{X}_{2}.\cdot \cdot \cdot .{X}_{n}\right\}s$ where *n* is the number of features, and the target variable as y. For each tree ${T}_{i}$ in the ensemble, the algorithm recursively partitions the data into subsets at each node, selecting the feature and split value that minimizes the mean squared error (MSE) of the target variable y. The final prediction $\widehat{y}$ for a given input $X$ is then obtained by averaging the predictions from all the individual trees in the forest:

3 $$\widehat{y}=\frac{1}{N}\sum_{i=1}^{N}{T}_{i}(X)$$

where N is the number of trees in the forest. This ensemble approach not only enhances the model's robustness and generalization but also allows for the calculation of feature importance scores, revealing the significance of each feature in the prediction process. The power of RF lies in its ability to handle high-dimensional data, resist overfitting, and deliver reliable predictions in various regression applications^44^.

*LR*: Logistic regression stands as a statistical technique employed to model the likelihood of a binary outcome, typically associated with events like success or failure. It hinges on the utilization of the logistic function, a transformative tool that maps the linear combination of input variables into bounded values within the range of 0 to 1. The formula that encapsulates logistic regression can be succinctly stated as:

4 $$P(Y=1)=\frac{1}{1+{e}^{-({b}_{0}+{b}_{1}{X}_{1}+{b}_{2}{X}_{2}+\dots +{b}_{n}{X}_{n})}}$$

In this equation, $P(Y=1)$ signifies the probability of the binary outcome assuming the value of 1, which in common scenarios corresponds to success. Key elements include ${b}_{0}$ , the intercept, and ${b}_{0}.{b}_{1}. \cdot \cdot \cdot \cdot$ the coefficients aligned with the input variables ${X}_{0}.{X}_{1}. \cdot \cdot \cdot \cdot$ It's worth noting that $e$ represents the base of the natural logarithm. The logistic function's elegance lies in its ability to confine the output to a probability scale between 0 and 1, a fundamental characteristic that renders logistic regression particularly suitable for classification tasks^45^.

*SVR*: is one of the most efficacious ML models for classification and regression. This method minimizes the structural risk by finding the most suitable decision function^46^. Briefly, using kernel functions, SVR can manage regression in high-dimensional datasets. Considering input and output vectors as X (from a ${R}^{N}$ input dataset) and Y such $\left\{\left(X.Y\right).X\in {R}^{N}. Y\in R\right\}$, respectively, SVR creates a linear function:

5 $$y={\omega }^{T}\phi \left(X\right)+c$$

With $\omega$ and c are the weight matrix and the bias value, respectively. The feature point X corresponding to a data point is mapped from the initial space to the hyperspace, which may be defined as $\sum {a}_{i}k\left(X.{X}_{i}\right)=const$. Where $k\left(X.{X}_{i}\right)$ is the kernel function and ${a}_{i}$ are non-negative Lagrangian coefficients. Depending on the type of kernel function, the performance of SVR may be changed. In this paper, SVR has been trained with Gaussian or RBF kernels, both linear and polynomial, and the best mode that results from the practice of non-linear RBF kernel functions is considered. The best kernel function is RBF kernel functions, or $k\left({X}_{i}.X\right)={\text{exp}}(\frac{-{\Vert {X}_{i}-X\Vert }^{2}}{2{\sigma }^{2}})$.

### Model evaluation

The absolute errors (AE) and the Mean square errors (MSE) were calculated via the following formulas to assess the performance of ML models:

6 $$AE= \left|{\widehat{y}}_{i}-{y}_{i}\right|$$

7 $$MSE=\frac{\sum_{i=1}^{n}\left|{\widehat{y}}_{i}-{y}_{i}\right| }{n}$$

As ${\widehat{y}}_{i}$ is the predicted target (size, E.E.%, or D.L.%) value; ${y}_{i}$ is the experimental value obtained from literatures. Total number of datasets in each target is shown with n.

### Feature Engineering: selection and reduction using LASSO

LASSO was first introduced by Robert Tibshirani, is a strong model for regularization and feature selection^47^. In this technique, the absolute value of the parameters has to be less than a fixed value (known as the upper bound) using a determined constraint in the method. In this regard, via a shrinking (regularization) process, the regression variables coefficients are penalized; some of them shrink to zero. Finally, non-zero coefficients are chosen to be part of the model.

This method aims to minimize the prediction error. In this method, λ determines the penalty, and with its larger values, the coefficients are dimensionality lowers down to zero. A higher λ value needs a greater number of coefficients that should be shrunk. However, its zero value gives rise to an Ordinary Least Squares (OLS) regression. LASSO predictions are very accurate since the bias doesn’t change during the shrinking process. Additionally, by eliminating irrelevant variables, LASSO increases the interpretability of the model as well as decreases the overfitting. Regarding to the advantages, LASSO has been chosen for the feature selection task.

### Supplementary Information

Supplementary Table 1.

## Data Availability

The data that support the findings of this study are gathered from published papers during last decade. Data will be available from the authors upon reasonable request.
