# Supplementary material for: Machine learning assisted exploration of the influential parameters on the PLGA nanoparticles
Source: Sci Rep. 2024 Jan 11;14:1114. doi: 10.1038/s41598-023-50876-w (PMC10784499; doi:10.1038/s41598-023-50876-w)
Supplement: Supplementary file 1 — Supplementary Table 1. [file 41598_2023_50876_MOESM1_ESM.docx]

Machine Learning Assisted Exploration of the Influential Parameters on the PLGA Nanoparticles

Sima Rezvantalab^1^, Sara Mihandoost^2^, Masoumeh Rezaiee^1^

^1^ Chemical Engineering Department, Urmia University of Technology, Urmia 57166‑419, Iran;

^2^ Electrical Engineering Department, Urmia University of Technology, Urmia 57166‑419, Iran;

Table SI outlines the prediction of selected method for each target using only one input feature at a time, and R-squared (an evaluation parameter ranging from 0 to 1, where R-squared = 1 indicates a model that can predict the output close to the actual value) was calculated for each prediction. As observed, the obtained results align with the LASSO results (Figure 4), indicating consistency in the predictive performance. The slight variations can be attributed to the interactions between different features when LASSO employs multiple features simultaneously to predict the target variable. These interactions may introduce additional complexity and contribute to the differences observed between the single-feature predictions and the LASSO model's predictions.

| Table S1. **Analyzing the effects of each input feature on each target individually in terms of r^2^** | | | | | | | | | |
| --- | --- | --- | --- | --- | --- | --- | --- | --- | --- |
| Target | ML technique | Method | Solvent 1 | Solvent 2 | PLGA Mw | LA/GA | PEG | PEG Mw | PVA |
| size | SVR | 0.417 | 0.171 | 0.003 | 0.286 | 0.312 | 0.495 | 0.160 | 0.312 |
| EE% | SVR | 0.131 | 0.092 | 0.003 | 0.300 | 0.131 | 0.002 | 0.007 | 0.022 |
| DL% | LR | 0.102 | 0.182 | 0.068 | 0.091 | 0.155 | 0.410 | 0.004 | 0.203 |
